# Mechanisms underlying fatigue: a voxel-based morphometric study of chronic fatigue syndrome

**DOI:** 10.1186/1471-2377-4-14

**Published:** 2004-10-04

**Authors:** Tomohisa Okada, Masaaki Tanaka, Hirohiko Kuratsune, Yasuyoshi Watanabe, Norihiro Sadato

**Affiliations:** 1 National Institute for Physiological Sciences, 38 Nishigonaka, Myodaiji, Okazaki, Aichi 444-8585, Japan; 2 Department of Physiology, Osaka City University Graduate School of Medicine, Osaka 545-8585, Japan; 3 Department of Health Science, Faculty of Health Science for Welfare, Kansai University of Welfare Sciences, Kashihara, Osaka 582-0026, Japan; 4 JST/RISTEX, 2-5-1 Atago, Minato-ku, Tokyo 105-6218, Japan

## Abstract

**Background:**

Fatigue is a crucial sensation that triggers rest, yet its underlying neuronal mechanisms remain unclear. Intense long-term fatigue is a symptom of chronic fatigue syndrome, which is used as a model to study the mechanisms underlying fatigue.

**Methods:**

Using magnetic resonance imaging, we conducted voxel-based morphometry of 16 patients and 49 age-matched healthy control subjects.

**Results:**

We found that patients with chronic fatigue syndrome had reduced gray-matter volume in the bilateral prefrontal cortex. Within these areas, the volume reduction in the right prefrontal cortex paralleled the severity of the fatigue of the subjects.

**Conclusion:**

These results are consistent with previous reports of an abnormal distribution of acetyl-L-carnitine uptake, which is one of the biochemical markers of chronic fatigue syndrome, in the prefrontal cortex. Thus, the prefrontal cortex might be an important element of the neural system that regulates sensations of fatigue.

## Background

Chronic fatigue is common and is reported in more than 20% of people seen in primary care [1]. However, the neural substrates of chronic fatigue are not well understood. For clinical use, central fatigue is defined as difficulty in the initiation of, or the ability to sustain, voluntary activities [2]. Central fatigue, in contrast with neuromuscular or peripheral fatigue, represents a failure to complete physical and mental tasks that require self-motivation and internal cues, in the absence of demonstrable cognitive failure or motor weakness [3]. Based on this definition, Chaudhuri and Behan [2] proposed a conceptual model for central fatigue. The work output of voluntary activity depends on the applied voluntary effort, which is controlled by motivational input and perceived effort via feedback from motor, sensory and cognitive systems. Hence, any dissociation between the level of internal input (motivational and limbic) and that of the perceived effort from applied voluntary effort results in the sense of fatigue. Assuming that pathological fatigue is an amplified sense of the normal (physiological) fatigue induced by changes in the variables regulating work output, clinical studies of fatigue disorders can provide clues regarding the neural substrates of fatigue. Symptoms of lesions in the pathways of arousal and attention, such as the reticular and limbic systems, and the basal ganglia, generally include pathological fatigue [2]. Fatigue can also be the primary symptom of a disease itself – this is the case in chronic fatigue syndrome (CFS), which might therefore prove to be a good model for studying the mechanisms underlying fatigue sensation.

CFS is a clinically defined condition characterized by severe disabling fatigue and a combination of symptoms, the prominent features being self-reported impairments in concentration and short-term memory, sleep disturbances and musculoskeletal pain [4]. The diagnosis of CFS can be made only after alternative medical and psychiatric causes of chronic fatigue have been excluded [4]. Recent studies found biochemical and genetic characteristics in CFS patients, such as a decreased concentration of serum acetyl-L-carnitine [5], a serotonin-transporter gene-promoter polymorphism [6], and autoantibodies against the muscarinic cholinergic receptor [7]. Among these, administration of L-carnitine, which is the precursor of acetyl-L-carnitine, is known to improve the clinical status of CFS patients [8]. In the brain, the acetyl moiety of acetyl-L-carnitine is utilized mainly for the biosynthesis of L-glutamate [9]. In CSF patients, a significant decrease in the uptake of acetyl-L-carnitine was found in several regions of the brain, including the prefrontal (Brodmann's area (BA) 9/46d), temporal (BA21 and 41), and anterior cingulate (BA24 and 33) cortices and cerebellum [9]. However, whether such focal cortical hypofunction is due to an anatomical abnormality has not yet been investigated. We hypothesize that there might be regions with explicit anatomical abnormalities that correlate with the severity of fatigue. To measure the reduction in gray-matter volume, we conducted voxel-based morphometry with high-resolution magnetic resonance imaging (MRI) [10,11].

## Methods

Sixteen CFS patients (aged 24–46 years; average age 34.0 years; 10 men and 6 women) and 49 age-matched healthy control subjects (aged 21–47 years; average age 34.4 years; 27 men and 22 women) were enrolled in the study. They were recruited from the outpatient fatigue clinic in Osaka University Hospital (HK's special clinic) where more than 430 CFS patients, who met the diagnostic criteria of CFS [4], are being followed. The protocol was approved by the ethical committee of the National Institute for Physiological Sciences, Japan, and all subjects gave their written informed consent for the study. The periods of CFS lasted between 10 and 244 months, and the mean duration was 69.8 months (Table 1). All CFS patients were unable to carry out normal activities or actively work for several days a week because of severe general fatigue at the time of diagnosis. The severity of fatigue was measured using self-reported ratings based on daily activities (performance status; Table 2). A detailed neurological examination, the time course of the patients' signs and symptoms, and additional MRI (for 7 out of 16 patients) made the diagnosis of multiple sclerosis (MS) unlikely. The characteristics of the patients are shown in Table 1. To compare brain volumes, high-resolution anatomical images were acquired using a 3 Tesla MR scanner (Allegra, Siemens, Erlangen, Germany). A three-dimensional structural MRI was acquired for each subject using a T1-weighted magnetization-prepared rapid-gradient echo sequence (repetition time, 1970 ms; echo time, 4.3 ms; inversion time, 990 ms; number of excitation, 1; flip angle, 8°; matrix size, 256 × 256; field of view, 210 × 210 mm) yielding 160 sagittal slices with a slice thickness of 1.2 mm and an in-plane resolution of 0.82 mm.

**Table 1:** Patient Characteristics

Patient number	Age (years)	Duration (months)	PS	Difficulty in thinking	Inability to concentrate
1	39	132	8	2	2
2	33	56	8	2	1
3	26	10	4	2	2
4	31	37	2	1	1
5	30	36	8	2	2
6	27	42	5	1	2
7	27	100	7	1	1–2
8	27	153	8	2	2
9	37	17	4	1	1
10	46	244	2	2	2
11	24	10	4	1	1
12	34	10	7	2	2
13	36	131	7	2	2
14	35	14	6	1	1
15	46	56	8	2	2
16	45	69	7	2	2

Level of fatigue, difficulty in thinking, inability to concentrate and depression are rated as follows: 2, severe; 1, mild; 0, none. PS, performance status at MRI examination.

**Table 2:** Performance-status scores for evaluating the severity of fatigue in CFS patients.

Scores	Condition
0	No complaints; able to carry on normal activity without fatigue.
1	Able to carry on normal activity, but sometimes feels fatigue.
2	Able to carry on normal activity or to do active work with effort; requires occasional rest.
3	Several days a month, unable to carry on normal activity or to do active work; requires rest at home without work.
4	Several days a week, unable to carry on normal activity or to do active work; requires rest at home without work.
5	Unable to carry on normal activity or to do active work at all, although able to perform light tasks; requires rest at home without work for several days a week.
6	Requires rest without work at home for over one-half of a week; able to do light tasks in good health.
7	Unable to carry on normal activity or to do light tasks at all; able to care for self without assistance.
8	Remains in bed for more than one-half of each day; able to care for self to some extent, but requires frequent assistance.
9	Unable to care for self; must remain in bed with day-long assistance.

Voxel-based morphometry (VBM) [12] was performed using SPM2 for image processing and was analyzed with SnPM99 [13] implemented in MATLAB 6.1 (MathWorks, Natick, MA, USA). VBM is a fully-automated whole-brain morphometric technique that detects regional structural differences between groups on a voxel-by-voxel basis. Briefly, images were segmented into gray matter, white matter, cerebrospinal fluid and skull/scalp compartments, then normalized to standard space and re-segmented. Any volume changes induced by normalization were adjusted [10,11]. The spatially normalized segments of gray and white matter were smoothed using a 12-mm full-width half-maximum isotropic Gaussian kernel. Statistical analysis of regional differences between groups was performed using a permutation test for decreased probability of a particular voxel containing gray or white matter. Potential confounding effects of age, sex and whole segment (gray or white matter) volume differences were modeled, and the variances attributable to them were excluded from the analysis [11,14,15]. The significance levels for statistics estimated by 500 nonparametric randomization and permutation tests were set at P = 0.05, corrected for multiple comparisons. Within the areas showing a significant volume reduction in patients, linear correlates between volume reduction and the degree of fatigue were examined under the threshold of P < 0.005.

## Results

**Figure 1:**
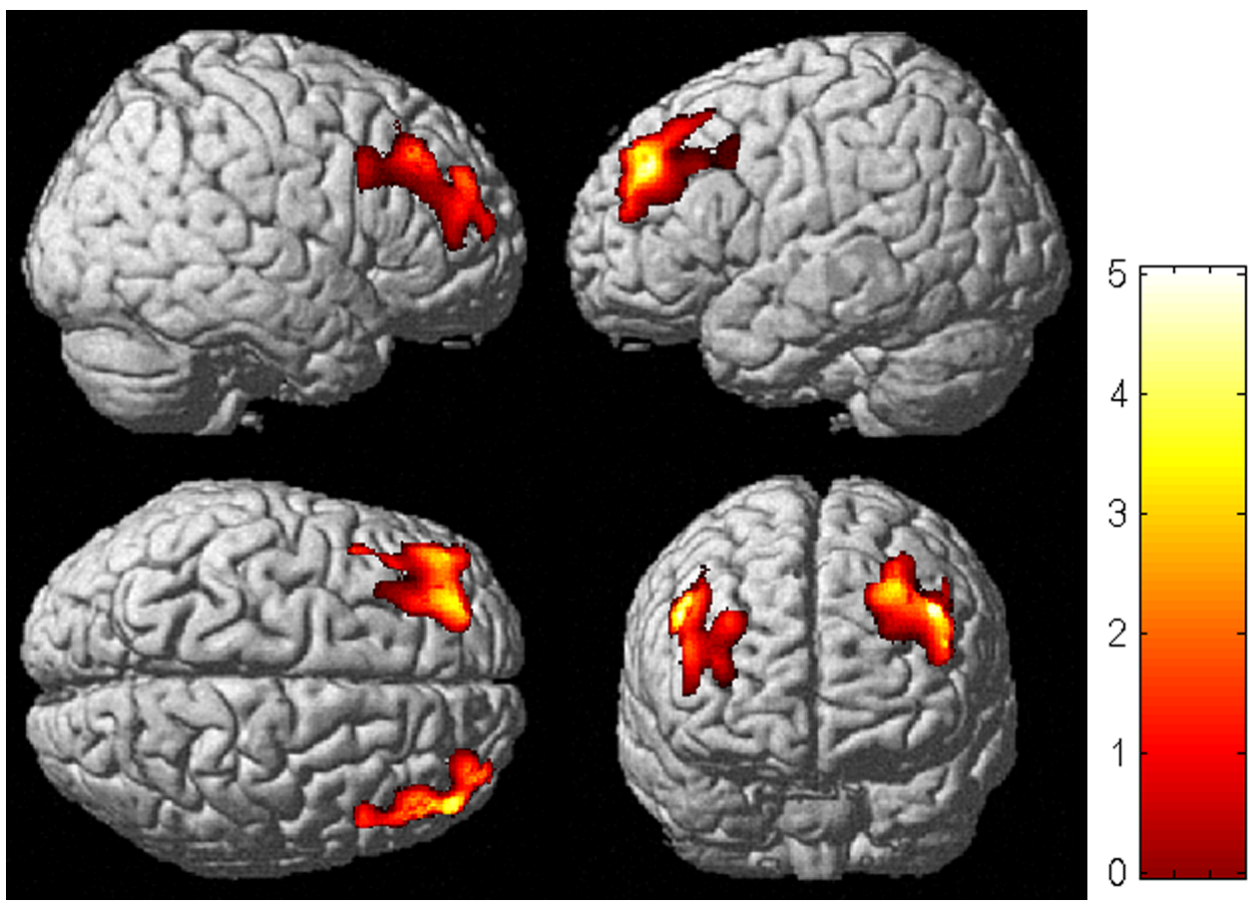
Regional differences between CFS patients and controls. Areas with significantly reduced gray-matter densities in the CFS patients were located at bilateral prefrontal areas, which were surface rendered onto the high-resolution MRI. The colored bar indicates the t-values.

**Figure 2:**
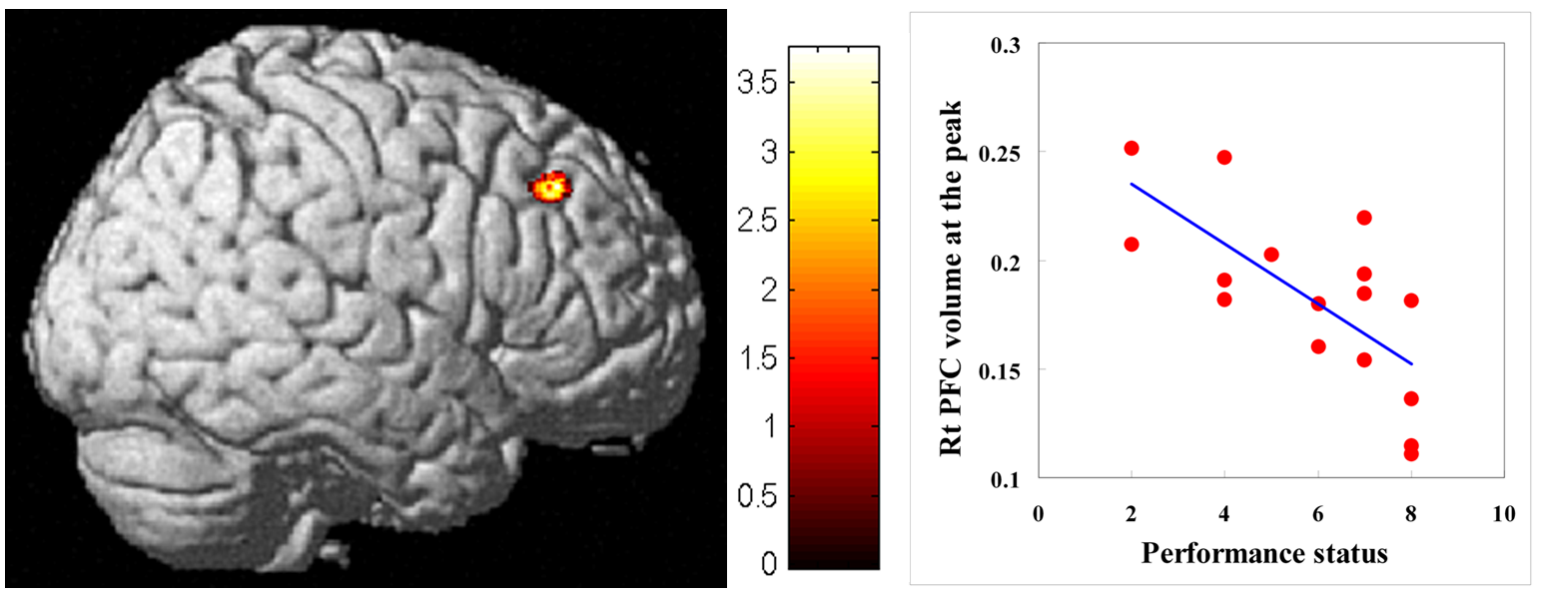
(Left) Correlations between volume and the performance status of CFS patients in the right prefrontal cortex (BA9; Talairach's coordinates: x = 48, y = 32 and z = 41). The colored bar indicates the t-values. (Right) Gray-matter volumes of CFS patients at the voxels of maximum correlation (r = 0.71) plotted against the performance status. The linear-regression line is plotted in blue. a.u., arbitrary units; Rt PFC, right prefrontal cortex.

We observed a significant reduction in gray-matter volume in the bilateral prefrontal areas of CSF patients (Figure 1). The affected areas extended from BA8 to 9 in the right cerebral hemisphere, and from BA9 to 11 in the left. In comparison with healthy controls, there was an average of 11.8% volume reduction in CSF patients. Within these areas, there was a significant negative correlation between the gray-matter volume of the right prefrontal cortex and the performance status of the CFS group (r^2 ^= 0.46, P = 0.004; Figure 2). This relationship was confirmed using Spearman's rank-correlation coefficient (P = 0.004). In this area, the gray-matter volume was reduced by 16.9% for patients compared with controls. No significant atrophy was observed in the white matter of CFS patients.

## Discussion

The present study provides the first report of focal gray-matter atrophy in the prefrontal cortex of CFS patients. Previous MRI studies of CFS revealed non-specific abnormalities: hyperintense small punctuated subcortical white-matter foci were observed predominantly in the frontal lobes [16] and their prevalence did not differ from an age-matched control group [17,18]. Ventricular enlargement was also reported [19]. Usually, MRI abnormalities in CSF patients cause the physician to conclude that the symptoms might be secondary to another medical condition [20].

Prefrontal pathology has been reported in MS with pathological fatigue [21]. Roelcke and colleagues [21] reported that MS patients with fatigue had a reduction of the cerebral metabolic rate of glucose (CMRGlu) in the bilateral prefrontal areas compared with MS patients without fatigue. Moreover, scores on the fatigue-severity scale were inversely related to CMRGlu levels in the right prefrontal cortex, suggesting that fatigue in MS is associated with prefrontal dysfunction due to the demyelination of frontal white matter [21]. Although the Talairach's coordinates reported by Roelcke and colleagues (x = 18, y = 42 and z = 20) were more medial and ventral than those observed here (x = 48, y = 32 and z = 41), both results suggest that prefrontal hypofunction might underlie pathological fatigue. Although MS should be excluded in the diagnosis of CFS, as in the present study, the similar clinical manifestations of the illnesses suggest that a common pathogenesis underlies the symptoms of fatigue in both disorders. This speculation is supported by the fact that the administration of L-carnitine, which improves fatigue in CFS patients, was effective for treating fatigue in MS patients [22].

In the present study, right dorsolateral prefrontal-cortex atrophy was significantly correlated with the severity of fatigue, as measured by the performance-status scores. As the performance status rates the daily activities that trigger or aggravate fatigue, this correlated volume reduction might reflect a functional deficiency that makes patients susceptible to fatigue.

A single site in the dorsolateral prefrontal cortex revealed the parallel between volume reduction and fatigue severity. This does not necessarily mean that it is fatigue-specific; instead, this area might be the part of the network that, when functioning sub-normally, results in pathological fatigue. Fatigue is also a symptom of diseases that affect the basal ganglia, and that interrupt the connection between the prefrontal cortex and thalamus [3]. Hence, frontal-subcortical circuits might be important contributors to the sense of fatigue.

The dorsolateral prefrontal cortex has dense widespread subcortical and cortical connections [23]. A series of parallel frontal-subcortical circuits have been described that link specific regions of the frontal cortex to the striatum, globus pallidus and thalamus [24]. These originate in the prefrontal cortex, project into the striatum (caudate, putamen and ventral striatum), connect to the globus pallidus and substantia nigra, and from there connect to the thalamus. There is then a final link back to the frontal cortex in each circuit, forming a closed loop [25]. Corticostriatal and thalamocortical connections use excitatory glutamatergic projections [25]. Frontal-subcortical circuits serve as organizational axes, integrating related information from widespread areas of the brain and mediating diverse behaviors. The three principal behaviorally-relevant circuits originate in the dorsolateral prefrontal, orbitofrontal and anterior cingulate cortices [26]. The marker behaviors specific to each circuit are executive dysfunction (dorsolateral prefrontal-subcortical circuit), disinhibition (orbitofrontal-subcortical circuit) and apathy (medial frontal-subcortical circuit), respectively [26]. Hence, these circuits are capable of concurrent participation in separate functions, including motor, cognitive and limbic processing [3].

The dorsolateral prefrontal cortex also has widespread reciprocal corticocortical connections with posterior temporal, parietal and occipital association areas [23]. Furthermore, at the level of the frontal lobes, the orbitofrontal, anterior cingulate and dorsolateral prefrontal cortices are linked to each other without cross connections at subcortical levels [26]. Therefore, the dorsolateral prefrontal cortex is poised to serve as a principal site for the integration of information.

These anatomical and functional characteristics of the frontal-subcortical circuits suggest that the large decrease in acetyl-L-carnitine uptake in the dorsolateral prefrontal, anterior cingulate and temporal cortices [9] represents hypofunction of the frontal-subcortical circuits. Furthermore, this decrease might be due to the remote effects of the pathology in the dorsolateral prefrontal cortex [27]. Recently, Fillippi et al. [28] underwent fMRI with MS patients with fatigue using simple motor task. They found inverse correlation between fatigue severity score and the task-related activity of the thalamus, concluding that fatigue could be secondary to dysfunction of corticosubcortical circuits. Thus, according to the model by Chaudhuri and Behan [3], hypofunction of the dorsolateral prefrontal cortex might interrupt the associated striato-thalamo-cortical loop, resulting in enhanced fatigability.

## Conclusions

The results of the present study suggest that the dorsolateral prefrontal cortex might be an important component of the neural substrates that regulate the sensation of fatigue.

## List of abbreviations

BA, Brodmann's area; CMRGlu, cerebral metabolic rate of glucose; CFS, chronic fatigue syndrome; MRI, magnetic resonance imaging; VBM, voxel-based morphometry

## Competing interests

The author(s) declare that they have no competing interests.

## Authors' contributions

TO carried out the MRI scanning, data analysis and drafted the manuscript. MT conducted MRI scanning and participants' coordination. HK conducted the medical evaluation of the participants. YW and NS participated in the study design. All authors read and approved the final manuscript.

## Pre-publication history

The pre-publication history for this paper can be accessed here:


